# Nitric oxide (NO) elicits aminoglycoside tolerance in *Escherichia coli* but antibiotic resistance gene carriage and NO sensitivity have not co-evolved

**DOI:** 10.1007/s00203-021-02245-2

**Published:** 2021-03-07

**Authors:** Cláudia A. Ribeiro, Luke A. Rahman, Louis G. Holmes, Ayrianna M. Woody, Calum M. Webster, Taylor I. Monaghan, Gary K. Robinson, Fritz A. Mühlschlegel, Ian B. Goodhead, Mark Shepherd

**Affiliations:** 1 School of Biosciences, RAPID Group, University of Kent, Canterbury, CT2 7NJ UK; 2 Clinical Microbiology Service, East Kent Hospitals University NHS Foundation Trust, William Harvey Hospital, Ashford, Kent, TN24 0LZ UK; 3 Laboratoire National de Santé 1, Rue Louis Rech, L-3555 Dudelange, Luxembourg; 4 School of Science, Engineering & Environment, University of Salford, Lancashire, M5 4WT UK

**Keywords:** Aminoglycoside, *Escherichia coli*, Nitric oxide, Respiration, Antibiotic resistance

## Abstract

The spread of multidrug-resistance in Gram-negative bacterial pathogens presents a major clinical challenge, and new approaches are required to combat these organisms. Nitric oxide (NO) is a well-known antimicrobial that is produced by the immune system in response to infection, and numerous studies have demonstrated that NO is a respiratory inhibitor with both bacteriostatic and bactericidal properties. However, given that loss of aerobic respiratory complexes is known to diminish antibiotic efficacy, it was hypothesised that the potent respiratory inhibitor NO would elicit similar effects. Indeed, the current work demonstrates that pre-exposure to NO-releasers elicits a > tenfold increase in IC_50_ for gentamicin against pathogenic *E. coli* (i.e. a huge decrease in lethality). It was therefore hypothesised that hyper-sensitivity to NO may have arisen in bacterial pathogens and that this trait could promote the acquisition of antibiotic-resistance mechanisms through enabling cells to persist in the presence of toxic levels of antibiotic. To test this hypothesis, genomics and microbiological approaches were used to screen a collection of *E. coli* clinical isolates for antibiotic susceptibility and NO tolerance, although the data did not support a correlation between increased carriage of antibiotic resistance genes and NO tolerance. However, the current work has important implications for how antibiotic susceptibility might be measured in future (i.e. ± NO) and underlines the evolutionary advantage for bacterial pathogens to maintain tolerance to toxic levels of NO.

**Supplementary Information:**

The online version contains supplementary material available at 10.1007/s00203-021-02245-2.

## Introduction

For the last eight decades, antibiotics have been used to combat infectious diseases. However, their widespread use has led to the emergence of bacterial pathogens with increased resistance to antimicrobial agents, leading to an increase in mortality and morbidity. Recently, the emergence of multidrug-resistance (i.e. to three or more antibiotic classes) in Gram-negative pathogens has become a cause for concern, with several Gram-negative bacteria, including *E. coli*, found to be NDM-1 β-lactamase-producing (Walsh et al. 2011). These strains often carry many other resistant determinants resulting in extreme multidrug-resistant phenotypes (Yong et al. 2009). Of particular concern is bacteraemia caused by *E. coli*, where there are approximately 40,000 annual cases in England. Over one-third of these are caused by drug-resistant strains causing close to 5,000 deaths each year (Public Health England). An over-represented clonal group is *E. coli* ST131, a uropathogen first identified in 2008 (Nicolas-Chanoine et al. 2008) that is now globally disseminated in both hospital and community settings and has a high incidence of CTX-M-15 extended-spectrum β-lactamase (ESBL)-producing isolates (Croxall et al. 2011; Nicolas-Chanoine et al. 2014). Given the apparent ease with which multidrug-resistant *E. coli* are able to spread throughout the human population, it is important to understand elements of the host environment and bacterial physiology that could potentially subvert the efficacy of antibiotics. Indeed, the ability of a bacterium to respire has a profound impact upon antibiotic function and is, therefore, a major focus of the current study. A recent study has shown that the abolition of aerobic respiration (reviewed in (Stokes et al. 2019)), dramatically reduces the toxic effects of bactericidal antibiotics (Lobritz et al. 2015). It, therefore, seems reasonable that a potent respiratory inhibitor such as a nitric oxide (NO), which is produced by the host immune system during infection, would also impair antibiotic efficacy.

It is well-known that bactericidal antibiotics can induce cell death via activation of pathways that result in oxidative damage via elevation of aerobic respiratory rates (Dwyer et al. 2007, 2014; Foti et al. 2012), with superoxide production implicated as a key intermediate: superoxide causes destabilisation of iron-sulphur complexes (Imlay and Fridovich 1991; Keyer and Imlay 1996) which causes iron dysregulation and, more importantly, leaching of ferrous iron. Subsequent reactions of leached ferrous iron reduce hydrogen peroxide to toxic hydroxyl radicals via the Fenton reaction (Imlay et al. 1988). Although different bactericidal antibiotics have different modes of action, Kohanski and colleagues (Kohanski et al. 2007, 2010) identified that most are able to induce the formation of hydroxyl radicals which is thought to be linked to the aerobic respiratory chain. However, for aminoglycosides, there is significant evidence to support a link between the proton motive force and antibiotic toxicity that is independent of ROS formation: uptake of positively charged aminoglycosides is linked to the electrical component of the proton motive force, as transport into the cell is thermodynamically favourable as the charge component of the PMF will be dissipated upon antibiotic entry (Taber et al. 1987; Farha et al. 2018).

**Fig. 1:**
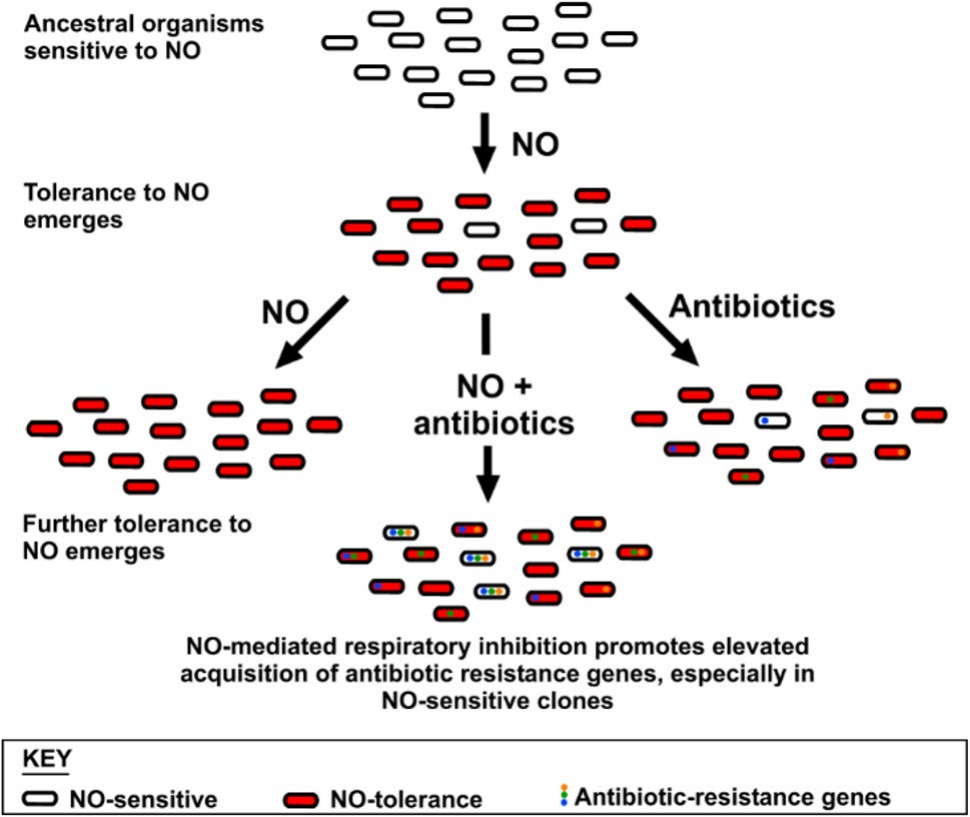
Initial hypotheses for the co-evolution of NO sensitivity and antibiotic resistance gene carriage

Aside from the potential role in disrupting the potency of antibiotics, the primary function of NO in the immune response is to act as a toxic antimicrobial to combat infection. Cellular targets for NO include thiols, metal centres, haem cofactors, and cysteine residues of proteins that are susceptible to *S*-nitrosylation (Fang 1997; Keszler et al. 2010). Bacteria have encountered NO in the gut and at the local site of infection for millennia, and have predictably evolved a variety of mechanisms to circumvent this toxicity (Shepherd et al. 2016), including detoxification systems to convert NO to less toxic substances, NO-tolerant respiratory oxidases, and mechanisms to repair damaged iron sulphur clusters. Hence, an assumption made in the current study is that bacterial exposure to NO and the evolution of resistance to this toxic molecule pre-dates the widespread exposure to antibiotics and the resultant emergence of antibiotic resistance. Given that loss of aerobic respiratory complexes had previously been shown to diminish the efficacy of various antibiotics (Lobritz et al. 2015), it was anticipated that the respiratory inhibitor NO would also diminish antibiotic susceptibility. If this could be demonstrated, we hypothesised that hyper-sensitivity to NO may have arisen in bacterial pathogens as a mechanism to promote the acquisition of antibiotic-resistance through enabling cells to persist in the presence of toxic levels of antibiotic (Fig. 1). Hence, the current work sought to investigate the relationship between NO tolerance/sensitivity and carriage of antibiotic resistance genes and to characterise the impact of NO upon the efficacy of conventional antibiotics against *E. coli* clinical isolates.

## Materials and methods

### Bacterial isolates

Fifty *E. coli* blood culture isolates were collected from East Kent Hospitals University NHS Foundation Trust. Approval was sought from the Research Ethics Council in advance (ref: 12/SC/0673) for this fully-blinded study. *E. coli* CFT073 is a highly invasive UPEC strain and a causative agent of pyelonephritis (Mobley et al. 1990; Welch et al. 2002), *E. coli* 83972 is asymptomatic bacteriuria (ABU) strain capable of out-competing UPEC strains (Roos et al. 2006), and EC958 is a well-characterised O25: H4-ST131 multidrug-resistant urosepsis-causing strain (Totsika et al. 2013; Forde et al. 2014). EC958 Cm-cassette insertional mutant strains *norVW* and *hmp* have been described previously (Shepherd et al. 2016).

### Genomics analyses

Multilocus sequence typing (MLST) was performed using the Achtman approach as previously described (Maiden et al. 1998; Clark et al. 2012). A minimum spanning tree was created based on the MLST data using BioNumerics version 7.5 created by Applied Maths NV. Bacterial genomes were sequenced using an Illumina MiSeq instrument, and reads were assembled de novo using SPAdes Genome Assembler (Bankevich et al. 2012), after which the genomes were re-ordered with the software Mauve (v2.4.0) (Darling et al. 2004) and using the well-characterised genome *E. coli* MG1655 (NC_000913.3) as reference. The newly re-ordered genomes were annotated with Prokka (v1.12) (Seemann 2014). Pan-genome analysis was accomplished with Roary (Page et al. 2015), and the core genome obtained was used as input in FastTree (Price et al. 2009) to create a phylogenetic tree of all the isolates of the Kent Collection, and edited with iTOL v3 (Letunic and Bork 2016). Genomes were mined for virulence genes using ABRicate (v0.8) (https://github.com/tseemann/abricate) to search the VFDB database (Chen et al. 2005). Protein sequences were aligned with T- Coffee (Notredame et al. 2000; Di Tommaso et al. 2011) and the values for protein identity and similarity of each pairwise alignment were extracted with BioEdit v7.2.5. For phylogenetic group determination, the Clermont method was used (Clermont et al. 2013, 2015). Briefly, the genomic sequence of each isolate was concatenated in FASTA format and used for in silico PCR using Unipro UGENE platform (Okonechnikov et al. 2012) with the primer sequences used by Clermont et al*.* (Clermont et al. 2013). To predict antibiotic susceptibility using genomic data, genomes were mined for antibiotic resistance genes with ABRicate (https://github.com/tseemann/abricate) to search the ResFinder 2.1 database (Zankari et al. 2012). For in silico detection of aminoglycoside resistance, the protein sequences of the gyrase subunit A for all genomes were aligned with T-Coffee (Notredame et al. 2000; Di Tommaso et al. 2011) and chromosomal mutations known to elicit resistance (Weigel et al. 1998; McArthur et al. 2013; Jia et al. 2017) were manually detected.

### Antimicrobial susceptibility assays

The British Society for Antimicrobial Chemotherapy (BSAC) method for antibiotic susceptibility testing was used to prepare standardised inoculum (Andrews 2001, 2002) and for breakpoint values (i.e. antimicrobial threshold concentrations that define resistance/susceptibility). Antimicrobial susceptibility profiles of *E. coli* clinical isolates were obtained for amoxicillin (AMX) (Sigma), cefotaxime (CTX) (Sigma), chloramphenicol (CAP) (ThermoFisher), ciprofloxacin (CIP) (Fluka), meropenem (MEM) (Sigma), nitrofurantoin (NIT) (Sigma), and trimethoprim (TMP) (Sigma) using the disc diffusion method. Due to poor agar diffusion, the susceptibility of the clinical isolates to polymyxin E (PME) (Sigma) was determined using the minimum inhibitory concentration (MIC) method.

### Gentamicin dose response assays

Cultures of EC958 wild type were grown to stationary phase overnight in LB at 37 °C. 10 mL volumes of M9 media (16 g/L Na_2_HPO_4_.2H_2_O, 3 g/L KH_2_PO_4_, 0.5 g/L NaCl, 1 g/L NH_4_Cl, 0.24 g/L MgSO_4_, 0.01 g/L CaCl_2_, and 4 g/L glucose) supplemented with 0.1% casamino acids (w/v) were prepared in 50 mL conical flasks and inoculated with 1% (v/v) of the stationary phase cultures, and incubated at 37 °C, 180 rpm until mid-exponential phase. From these cultures, suspensions of 10^8^ CFU/mL were prepared in fresh M9 media supplemented with 0.1% casamino acids and exposed to GSNO (0 or 15 mM) or NOC-12 (0 or 1 mM) for 30 min at 37 °C, prior to incubation with different concentrations of gentamicin for 90 min. Serial dilutions were performed in 1 × PBS and cells were plated in triplicate on LB-agar plates. Colony counts were performed after overnight incubation at 37 °C. Two biological repeats were performed for each condition, with three technical repeats performed for each biological repeat (i.e. 6 in total).

### Nitric oxide susceptibility assays

Nitric oxide susceptibility was evaluated using a well diffusion assay. Briefly, cultures at mid-exponential growth were prepared in M9 minimal medium supplemented with 0.1% casamino acids, using a 1% inoculum of stationary phase aerobic culture prepared in LB medium at 37 °C. When mid-log phase was reached, cells were plated on M9 minimal agar medium using the pour plate technique. Six 6 mm diameter-wells were cut in the agar gel and 80 µL of 80 mM *S*-Nitrosoglutathione (GSNO), synthesised and quantified as previously described (Hart 1985), was added to each. Plates were incubated at 37 °C under aerobic, microaerobic (2% oxygen) or anaerobic conditions. For anaerobic growth, 50 mM fumarate was included in the agar. For microaerobic growth, oxygen levels were controlled using a Ruskinn Invivo 300 Hypoxia Workstation and for anaerobic growth, plates were incubated in anaerobic jars with an anaerobic gas pouch (Oxoid) and oxygen indicator (Oxoid). Zones of inhibition were measured after 16 h incubation, and data were analysed using a two-tailed unpaired Student’s *t*-test.

## Results

### Nitric oxide diminishes antibiotic efficacy

**Fig. 2:**
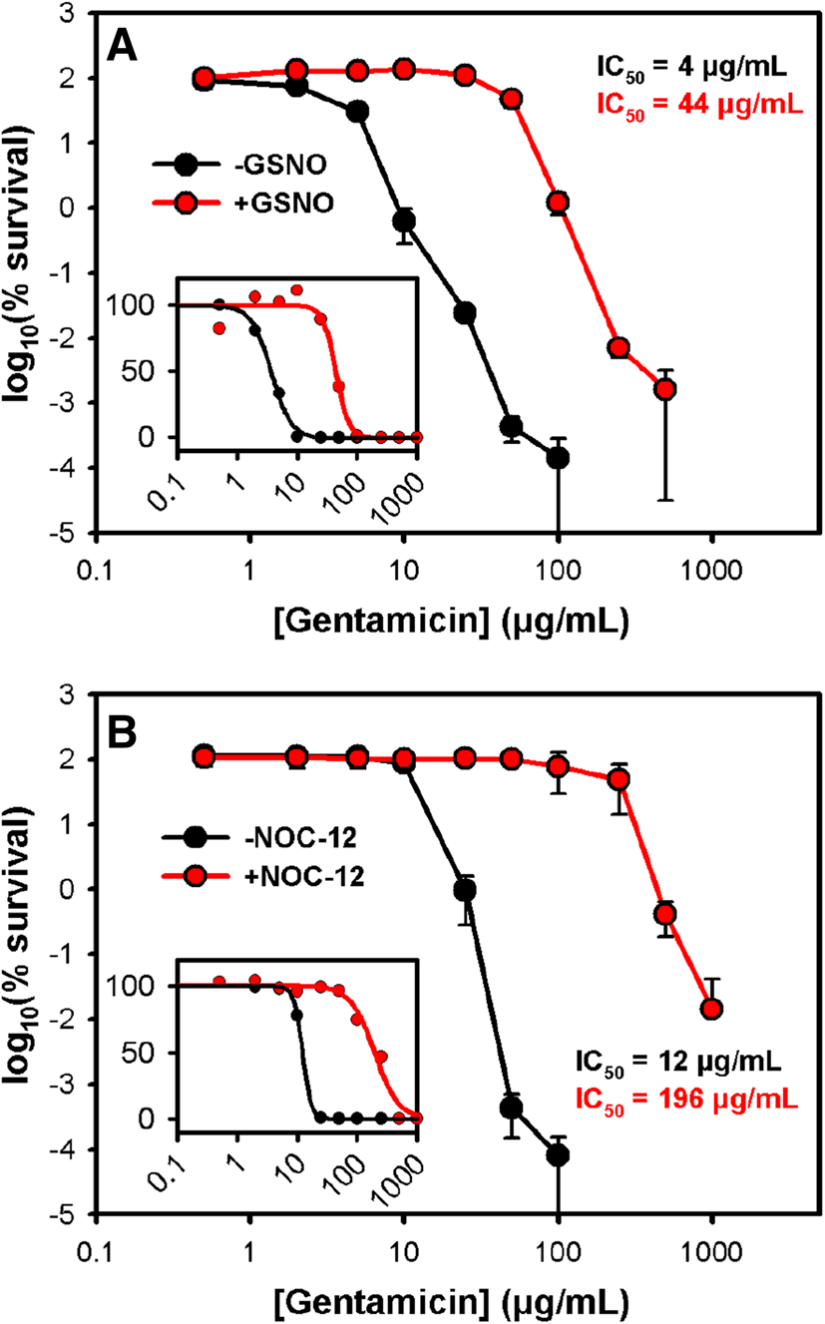
Nitric oxide releasers abrogate the lethality of gentamicin. Suspensions of 10^8^ cells/mL of *E. coli* EC958 in M9 minimal media supplemented with 0.1% casamino acids were exposed to 15 mM GSNO (**a**) and 1 mM NOC-12 (**b**) for 30 min, followed by incubation with different concentrations of gentamicin for 90 min. Serial dilutions were performed in PBS and plated on LB-agar. CFU/mL values were determined after overnight incubation at 37 °C. Data were fitted to a four parameter Hill equation for calculation of IC_50_ values, and magnitude changes were normalised to 100% for display on a linear y-axis (insets). Values shown represent mean ± SD from two biological repeats, each comprising three technical repeats

It has previously been demonstrated that NO protects *Salmonella enterica*, *Pseudomonas aeruginosa* and *Staphylococcus aureus* from aminoglycosides by blocking the energy-dependent phases of drug uptake (McCollister et al. 2011), although no detailed analyses were undertaken to measure the impact upon antibiotic IC_50_ values, and no work was undertaken on pathogenic *E. coli*. Hence, a gentamicin dose response killing experiment was conducted with the multidrug-resistant *E. coli* O25b:H4-ST131 (EC958) strain (Totsika et al. 2013; Forde et al. 2014) using the NO-donor GSNO. Pre-exposure of an exponentially-growing culture of EC958 to 15 mM GSNO for 30 min, prior to the addition of increasing gentamicin concentrations for 90 min, led to a dramatic increase in bacterial survival (Fig. 2a) with the IC_50_ increasing approximately tenfold for cells pre-exposed to GSNO. To confirm that this effect was due to the presence of NO, a similar experiment was performed with 1 mM NOC-12 in place of GSNO (Fig. 2b), which confirmed the same pattern with a slightly more pronounced effect: pre-treatment with NOC-12 resulted in a 16-fold increase in the IC_50_ of gentamicin. A GSNO-mediated increase in IC_50_ was also observed under anaerobic conditions (Fig. S1), and it was also noted that a switch to anaerobiosis elicited a > tenfold increase in the IC_50_ for gentamicin in the absence of GSNO.

### No detectable correlation exists between antibiotic-resistance gene carriage and nitric oxide susceptibility in E. coli clinical isolates

**Fig. 3:**
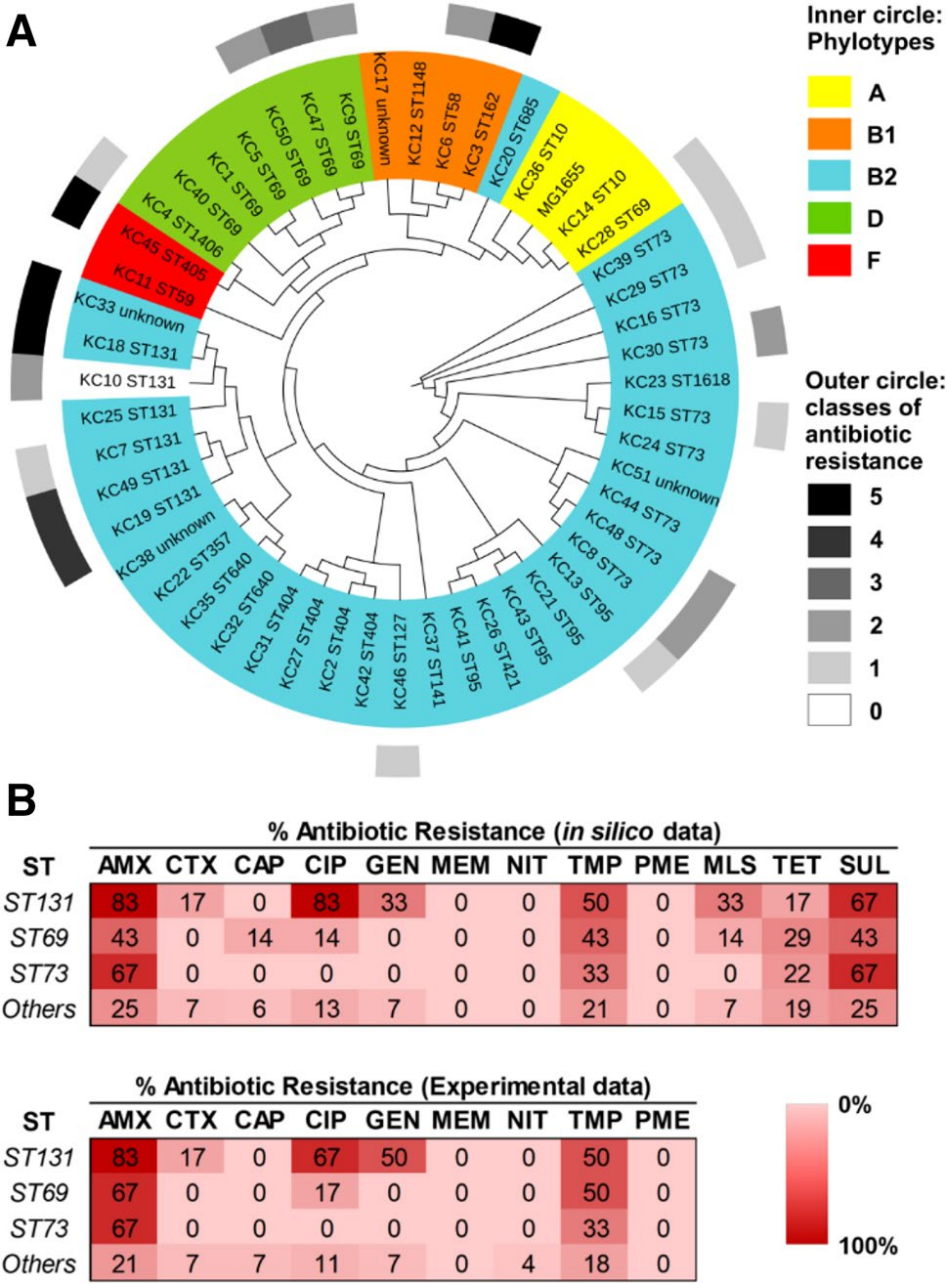
Antibiotic susceptibilities of *E. coli* phylogroups and sequence types. **a** Phylogenetic relationships of 50 *E. coli* clinical isolates were inferred using the core genome gene sequences of all isolates (compared to *E. coli* MG1655) obtained by pan-genome analysis with Roary. Phylogroups for each isolate were determined using in silico PCR, and each colour represents one phylogroup in the tree (inner circle). **b** Antibiotic resistance profiles were predicted using bioinformatics approaches and were also experimentally screened for antibiotic resistance using disc susceptibility testing (Andrews 2001, 2002) (the total number of antibiotic classes that each isolate is resistant to, based on disc susceptibility, is shown in the outer ring of (**a**)). ‘% antibiotic resistance’ is defined as the % of isolates within a sequence type that are resistant to a particular antibiotic. *ST* sequence type, *AMX* amoxicillin, *CTX* cefotaxime, *CAP* chloramphenicol; *CIP* ciprofloxacin, *Gen* gentamicin, *MEM* meropenem, *NIT* nitrofurantoin, *TMP* trimethoprim, *PME* polymyxin E, *MLS* macrolides, *TET* tetracycline, *SUL* sulfanilamides

To investigate the potential relationship between antibiotic-resistance gene carriage and NO-mediated respiratory inhibition, it was necessary to perform genomic and phenotypic analysis of *E. coli* clinical isolates. Fifty *E. coli* bacteraemia isolates were collected from the Clinical Microbiology Service at The William Harvey Hospital (Ashford, Kent) in a double-blinded study, and will be herein referred to as the Kent Collection (KC). MLST analysis performed on KC isolates identified a total of 23 unique sequence types (STs), with the most prevalent STs ST73, ST69, and ST131, accounting for 18%, 14%, and 12% of the collection, respectively (Fig. S2). Isolates were sequenced using the Illumina MiSeq platform, and phylogenetic analysis with Roary (Page et al. 2015) was used to perform phylogroup clustering based on core genomes (Clermont et al. 2013, 2015) (Fig. 3a). To characterise antibiotic susceptibility and resistance gene carriage in this collection, a combination of in silico and laboratory techniques were employed. This dual approach was undertaken as in silico techniques could be used to rapidly screen all isolates for resistance to many classes of antibiotic, and then the more labour intensive experimental laboratory approach was undertaken on a subset of the antibiotics to validate the in silico results. These analyses revealed that 14% of the collection exhibited multidrug resistance (i.e. resistant to three or more antibiotic classes), with three such isolates belonging to the ST131 clonal group (Fig. 3a, outer ring). Furthermore, 6% were CTX-M-15 ESBL-producing isolates, and susceptibility to amoxicillin, trimethoprim, and ciprofloxacin was found in 42%, 28%, and 16% of the isolates, respectively. Resistance data for the most prevalent STs, which are also the clonal groups with the highest degree of antibiotic resistance (i.e. ST131, ST69, ST73), are shown in Fig. 3b.

**Fig. 4:**
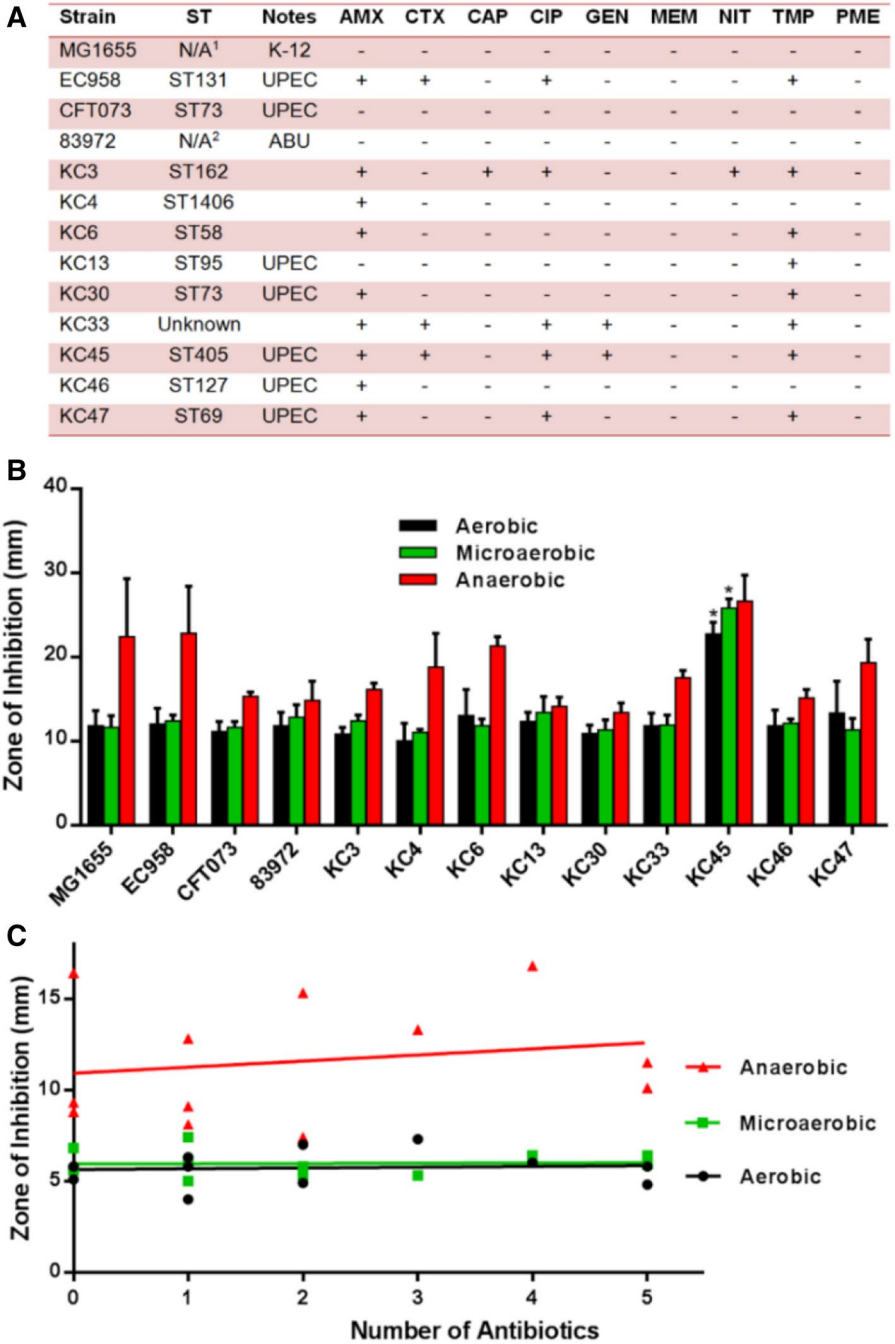
Antibiotic-resistant *E. coli* clinical isolates are not resistant/sensitive to NO. **a** Antibiotic resistance profiles of *the most resistant isolates in the KC collection* compared to MG1655, EC958, CFT073, and ABU strain 83972. *AMX* Amoxicillin, *CTX* Cefotaxime, *CAP* Chloramphenicol, *CIP* Ciprofloxacin, *MEM* Meropenem, *NIT* Nitrofurantoin, *TMP* Trimethoprim, *PME* Polymyxin E; (−) Sensitive; ( +) Resistant. ^1^ *N/A* Not Applicable. **b **NO susceptibility of antimicrobial-resistant isolates representative of STs, under different oxygen conditions. Values shown are the mean ± SD from three experiments. (*Student’s *t*-test unpaired two-tail *P*-value < 0.05). **c** Linear regression and correlation between GSNO susceptibility and antibiotic resistance. Correlation between GSNO susceptibility and the degree of antibiotic resistance (reflected by the number of antibiotics to which each isolate was experimentally resistant to) was calculated using the Pearson r for all strains in panels A and B except KC45 (Pearson *r* = 0.08, 0.04 and 0.19 for aerobic, microaerobic, and anaerobic conditions respectively. *P*-values of 0.79, 0.91, and 0.56 were obtained for aerobic, microaerobic, and anaerobic conditions, respectively). Linear relation between the two variables was assessed with linear regression, with *R*^2^ values of 0.007, 0.001, and 0.035 for aerobic, microaerobic, and anaerobic conditions, respectively)

**Fig. 5:**
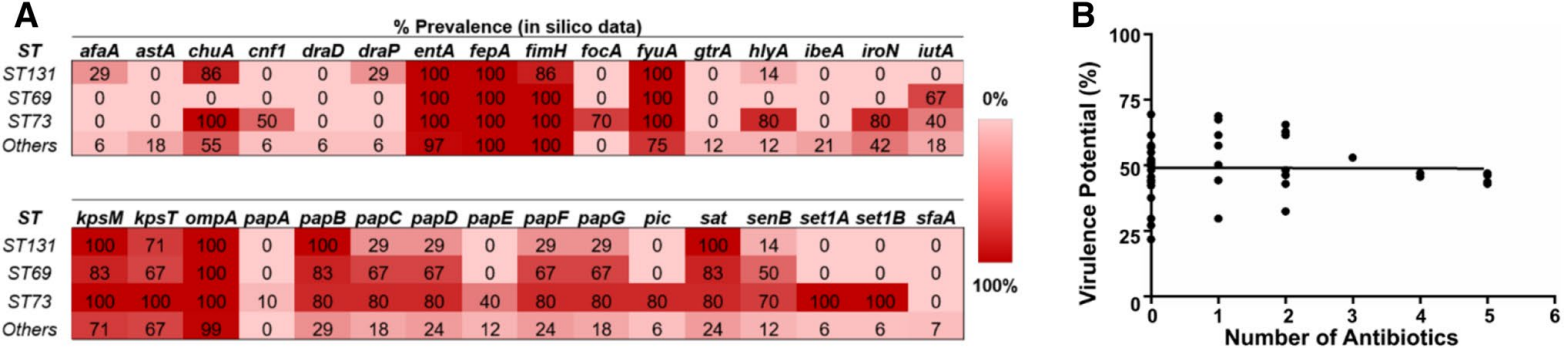
Virulence gene profiles of *E. coli* bacteraemia isolates: no correlation with antibiotic resistance. **a **50 clinical isolates were sequenced and virulence genes were identified using bioinformatics approaches. A selection of genes is shown for the most abundant sequence types in the collection. **b **The correlation between the virulence potential and the degree of antibiotic resistance (reflected by the number of different antibiotic classes to which each isolate was experimentally resistant to) was investigated by calculating the Pearson Correlation Coefficient (Pearson *r* = − 0.0097; *P*-value = 0.95). Virulence potential is calculated as the % of the 151 virulence genes identified that are carried in a given isolate. A relationship between the two variables was assessed using linear regression (*R*^2^ = 9.4 × 10^–5^). *ST* sequence type

To investigate potential correlations between antibiotic resistance and tolerance to NO, isolates that exhibited the highest degree of antibiotic resistance for each ST were selected (Fig. 4a), and susceptibility to the NO-releasing molecule GSNO was measured both in the presence (aerobic and microaerobic) and absence (anaerobic) of oxygen (Fig. 4b). Well-characterised *E. coli* clinical isolates EC958 (Totsika et al. 2013; Forde et al. 2014), CFT073 (Mobley et al. 1990; Welch et al. 2002) and 83,972 (Roos et al. 2006) were also analysed as control samples and known NO-sensitive mutant strains were confirmed to have larger zones of inhibition to validate the technique (Fig. S3). Out of the 13 strains tested, none of the isolates displayed a significant increase in NO tolerance compared to the control strains (Student’s unpaired two-tailed *t*-tests), and only one isolate (KC45) exhibited significantly higher susceptibility to NO than all other strains (*P*-value ≤ 0.001) under both aerobic and microaerobic conditions tested. However, further assessment of NO-sensitivity using the alternative NO-releasing compound NOC-12 revealed no sensitivity (Fig. S4), suggesting that this KC45 isolate is sensitive to a GSNO breakdown product other than NO. For this reason, the KC45 strain was omitted from the correlation analyses between antibiotic resistance and GSNO sensitivity for the different oxygen conditions, where no significant relationship was observed (Fig. 4c). To investigate potential correlations between antibiotic resistance and virulence gene carriage, genomic sequences were mined for virulence genes (Fig. 5a), although no significant correlation was found between the degree of antibiotic resistance and virulence profile from the Pearson Correlation Coefficient (Fig. 5b).

## Discussion

The antimicrobial properties of nitric oxide are well-known, and it has long been considered a potential alternative to antibiotics or for use in combination with antibiotics. However, given the anticipated potent inhibition of respiration in the presence of NO in aerobically-growing planktonic *E. coli* cells, it was hypothesised that NO would enhance tolerance to antibiotics (Fig. 1). Indeed, pre-exposure of *E. coli* EC958 with the NO-donors GSNO or NOC-12 prior to treatment with gentamicin dramatically increased (i.e. > tenfold increase in IC_50_) bacterial tolerance to this antibiotic (Fig. 2). In addition, GSNO was shown to offer significant protection to gentamicin in anaerobically-grown cells (Fig. S1), which suggests that there are cellular targets for GSNO other than respiratory oxidases that can influence tolerance to aminoglycosides. Interestingly, the IC_50_ for gentamicin increased by > tenfold when cells were grown anaerobically without GSNO, highlighting the dramatic influence that growth conditions can have upon antibiotic susceptibility. Given that NO is produced by the host immune system during infection, these data should change the way that antibiotic susceptibility is studied both during infection and in microbiological culture media and it would be particularly interesting to re-assess IC_50_ values for all antibiotics and all organisms via the BSAC method in the presence of NO for cells grown both aerobically and anaerobically.

**Fig. 6:**
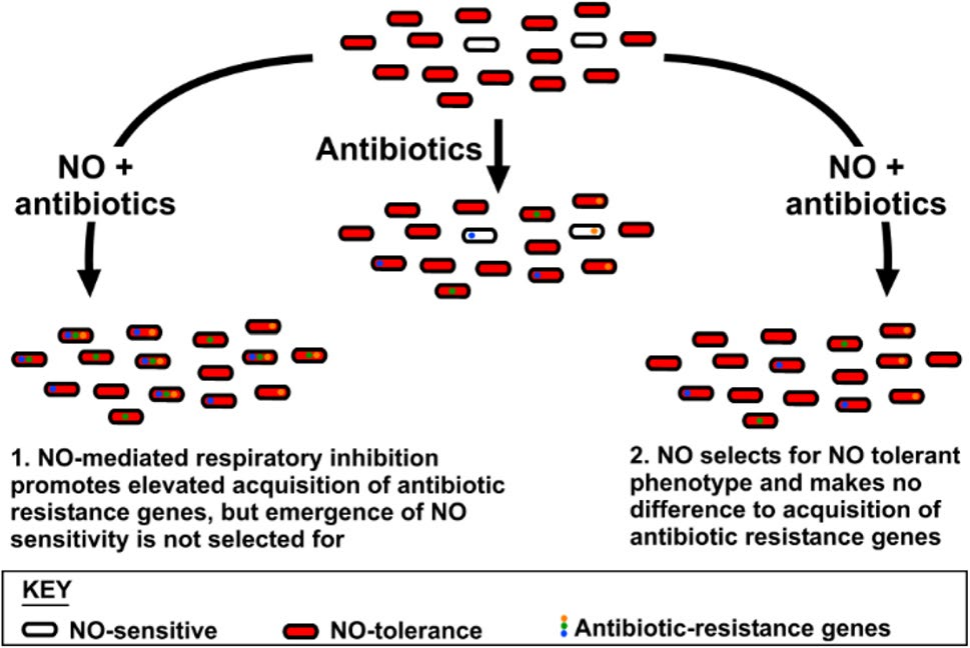
Alternative explanations to explain the relationship between NO tolerance and antibiotic resistance gene carriage. Two alternative explanations are provided to explain the absence lack of correlation observed in the current study between NO sensitivity and antibiotic resistance gene carriage

Given that both NO and antibiotics are encountered during infection, and that susceptibility to NO-mediated growth inhibition can prolong exposure of bacteria to antibiotics, it was hypothesised that hypersensitivity to NO could serve as an evolutionary driver for the acquisition of antibiotic resistance genes (Fig. 1). To investigate this, genomic characterisation of 50 *E. coli* bacteraemia isolates was undertaken and revealed the three most common STs to be ST73, ST69, and ST131 (Fig. 3), which have previously been shown to be comprised of multidrug-resistant strains (Croxall et al. 2011). In this collection, the ST131 isolates contained a greater incidence of antibiotic resistance compared to ST73, which is consistent with previous studies (Johnson et al. 2010; Forde et al. 2014; Nicolas-Chanoine et al. 2014b). Deletion of all three respiratory oxidase complexes in a non-pathogenic strain of *E. coli* has previously been shown to confer tolerance to antibiotics (Lobritz et al. 2015), so it was hypothesised that some bacterial isolates may have evolved sensitivity to the respiratory inhibitor NO, which could prolong survival in the presence of antibiotics and potentially influence the acquisition of antibiotic-resistance genes. However, only one of the drug-resistant clinical isolates (KC45) exhibited higher sensitivity to GSNO (Fig. 4b), and further experiments with the NO-releaser NOC-12 demonstrated a lack of sensitivity to NO (Fig. S4), suggesting that the KC45 strain is sensitive to an alternative breakdown product of GSNO other than NO itself (e.g. possibly glutathione). In fact, the KC45 and EC958 clinical isolates were both less sensitive to NOC-12 compared to the non-pathogenic MG1655 strain. Hence, the KC45 isolate was excluded from the correlation analysis (Fig. 4c), which is consistent with no relationship between NO sensitivity and antibiotic gene carriage. While elevated sensitivity to nitric oxide is likely to increase tolerance to antibiotics, a hypothesis presents itself that could explain the absence of a ‘NO sensitive’ phenotype in the KC collection: NO sensitivity provides a selective advantage during exposure to antibiotics, but the loss of fitness that accompanies an ‘NO sensitive’ phenotype is too detrimental to growth and survival outside this niche. Clearly, NO donors have a profound effect upon antibiotic susceptibility of the EC958 strain (Fig. 2), which possesses a full complement of intact NO tolerance machinery. This is consistent with the conclusion that NO is sufficiently potent to elicit similar effects against all *E. coli* strains, precluding the selection pressure for the emergence of NO sensitivity phenotype that is accompanied by a fitness cost as mentioned above. Hence, the hypothesis for co-evolution of NO sensitivity and antibiotic resistance gene carriage in Fig. 1 can be rejected and the current data can be explained by either of the two possibilities in Fig. 6: 1) NO-mediated respiratory inhibition promotes the elevated acquisition of antibiotic resistance genes but NO sensitivity is not selected for, or 2) NO has no impact upon acquisition of antibiotic resistance genes.

Previous work has demonstrated an inverse correlation between antibiotic resistance and virulence in *S. aureus* (Sapri et al. 2013) and there are many reports of resistance mechanisms affecting virulence (Beceiro et al. 2013), so it was of interest to screen this collection for correlations of this sort. However, no relationship between virulence and antibiotic resistance was observed in the KC collection studied herein (Fig. 5).

Given the direct toxic effects of NO upon bacterial cells (Fig. 2) and the ability of NO to disperse biofilms, NO-releasing compounds are potentially useful to treat bacterial infections where conventional antibiotics are no longer effective. Indeed, a combination of NO releasers and ciprofloxacin has previously been shown to be effective against *E. coli* and *P. aeruginosa* biofilms (de la Fuente-Núñez et al. 2013; Reffuveille et al. 2015). However, the current data show that NO has the opposite effect for gentamicin and planktonic *E. coli* cells, potentially due to NO-mediated respiratory inhibition diminishing the efficacy of the aminoglycoside gentamicin. More broadly speaking, diminished metabolic activity is becoming widely accepted to have a profound impact upon antibiotic susceptibility in general (Stokes et al. 2019), so the impairment of metabolic activity beyond respiratory metabolism caused by NO exposure (e.g. through destruction of iron-sulphur clusters and protein *S*-nitrosation) is likely to reduce the efficacy of a variety of antibiotic classes. While no strains with increased NO sensitivity were detected herein, no strains with increased NO tolerance were present either, suggesting that the entire collection would be resistant to gentamicin (and other antibiotics) in the presence of NO. With the potent effects that NO has upon antibiotic efficacy in clinical isolates in mind, it is clearly important to fully understand the combined effects of antibiotic treatments and nitrosative stresses, especially when devising new strategies to combat multidrug-resistant strains.

## Supplementary Information

Below is the link to the electronic supplementary material.

Supplementary file1 (DOCX 395 KB)
