# Supplementary material for: Nitric oxide (NO) elicits aminoglycoside tolerance in Escherichia coli but antibiotic resistance gene carriage and NO sensitivity have not co-evolved
Source: Arch Microbiol. 2021 Mar 7;203(5):2541–50. doi: 10.1007/s00203-021-02245-2 (PMC8205896; doi:10.1007/s00203-021-02245-2)
Supplement: Supplementary file 1 — Supplementary file1 (DOCX 395 KB) [file 203_2021_2245_MOESM1_ESM.docx]

**Supplemental Data**

Figure S1 - Nitric oxide releasers abrogate the lethality of gentamicin under anaerobic conditions. *E. coli* EC958 cells were grown in M9 minimal media supplemented with 0.1% casamino acids, glucose (2 g/L) and fumarate (40 mM). For aerobic growth, 50 mL cultures in 250 mL conical flasks were incubated at 37 ^o^C and 180 rpm. For anaerobic growth, media was sparged with nitrogen and cultures were grown statically at 37 ^o^C in sealed serum bottles. Suspensions of 10^8^ cells/mL were exposed to 15 mM GSNO for 30 min, followed by incubation with different concentrations of gentamicin for 90 min. Serial dilutions were performed in PBS and plated on LB-agar. CFU/mL values were determined after overnight incubation at 37°C. Data were fitted to a four parameter sigmoid for calculation of IC_50_ values, and magnitude changes were normalised to 100 % for display on a linear y-axis (inset). Values shown represent mean ± SD from two biological repeats, each comprising three technical repeats.

**

**

**Figure S2** **- Minimum spanning tree of 50 *E. coli* clinical isolates from Kent**. Number of partitions in each node indicates the total number of isolates with that sequence type (ST). Isolates with the same sequence type possess seven identical alleles. A single locus variant differs from the other sequence type by one allele; a triple locus variant differs by three alleles; and a more than triple locus variant differs by more than three alleles. The four unknown sequence types in the collection are shown as ‘?1’, ‘?2’, ‘?3’, and ‘?4’. The most prevalent STs ST73, ST69, and ST131, account for 18%, 14%, and 12% of the collection, respectively.


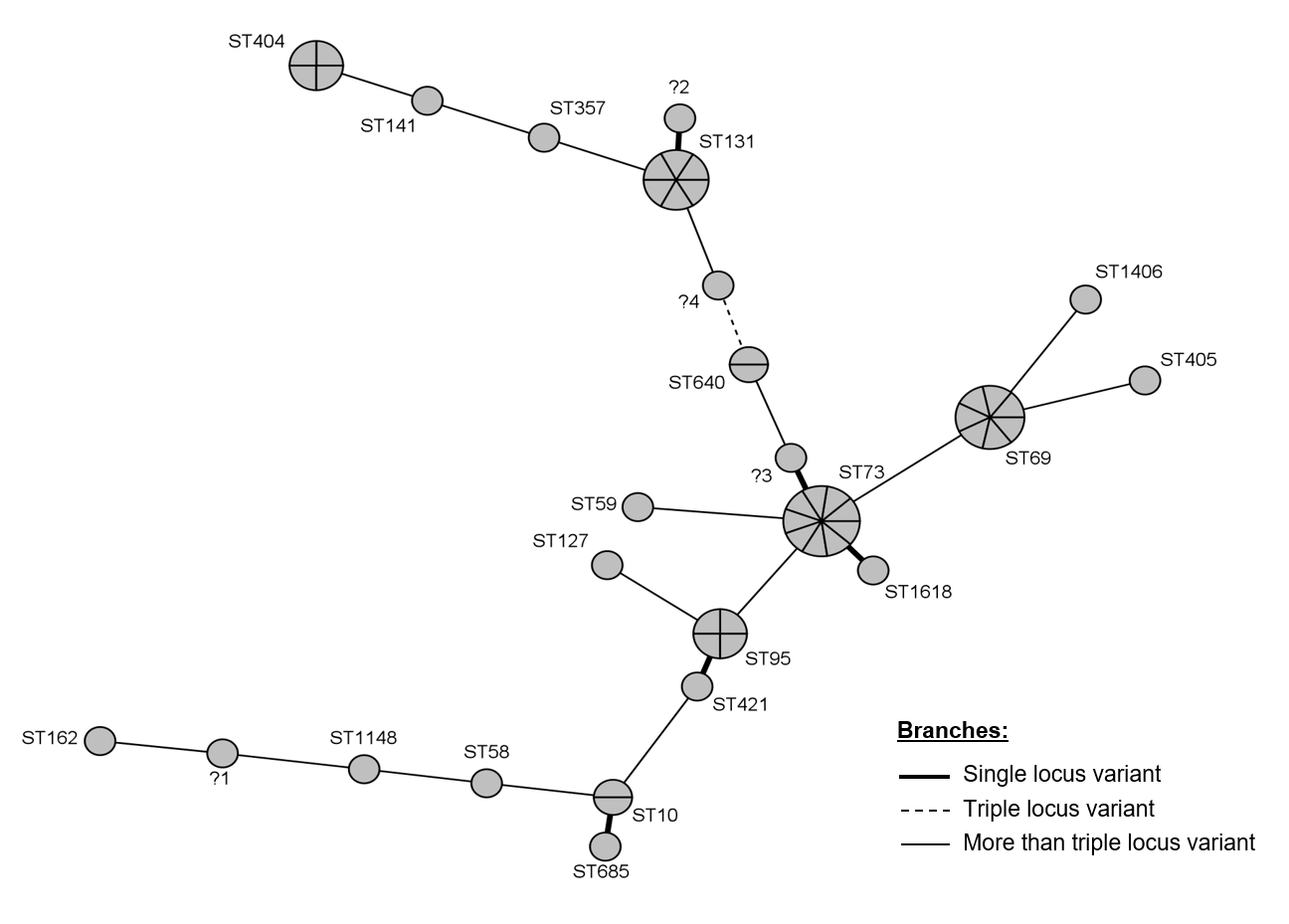


**Figure S3 – Validation of the GSNO disc diffusion method for NO sensitivity.** NO susceptibility of wild type and known NO-sensitive *hmp* and *norVW* strains were tested under different oxygen conditions. Loss of Hmp leads to elevated susceptibility to NO under aerobic and microaerobic conditions. As expected, loss of NorVW leads to elevated NO susceptibility under anaerobic conditions. Values shown are the mean ± SD from three experiments. (*Student’s *t*-test unpaired two-tail *P*-value < 0.05, compared to the corresponding data for the wild type strain).

**

**

**Figure S4 – Multidrug-resistant KC45 is not sensitive to the NO-releaser NOC-12.** NO-sensitivity was assessed in liquid medium using growth curves in the presence of the slow NO-releaser NOC-12 (t_1/2_= 100 min at 37^o^C, pH 7.4) as previously described (Mason et al. 2009; Shepherd et al. 2016). Briefly, cultures (200 µL) were grown in 96-well plates (37^o^C, 160 rpm) in M9 medium supplemented with casamino acids (0.1 %) and glucose (2 g/L). NOC-12 was suspended in 50 mM sodium phosphate pH 8.0 immediately before use and added to the culture when OD_600_ reached 0.04. Growth of *E. coli* MG1655 *(A),* EC958 *(B)*, and KC45 *(C)* were followed. Error bars represent SD values. All data points are mean values calculated from three biological repeats, each comprising three technical repeats. (*D*) Growth rates (expressed as doubling times) were calculated from OD_600_ readings for the 1.5 h following addition of NOC-12. Error bars represent SD values. (*: Student’s two-tailed unpaired *t*-test; *P*-value <0.05, compared to the ‘no treatment’ data for the same strain).

**

**
